# *Leishmania amazonensis* Arginase Compartmentalization in the Glycosome Is Important for Parasite Infectivity

**DOI:** 10.1371/journal.pone.0034022

**Published:** 2012-03-30

**Authors:** Maria Fernanda Laranjeira da Silva, Ricardo Andrade Zampieri, Sandra M. Muxel, Stephen M. Beverley, Lucile M. Floeter-Winter

**Affiliations:** 1 Departamento de Fisiologia, Instituto de Biociências, Universidade de São Paulo, São Paulo, São Paulo, Brazil; 2 Department of Molecular Microbiology, Washington University School of Medicine, St. Louis, Missouri, United States of America; State University of Campinas, Brazil

## Abstract

In *Leishmania*, *de novo* polyamine synthesis is initiated by the cleavage of L-arginine to urea and L-ornithine by the action of arginase (ARG, E.C. 3.5.3.1). Previous studies in *L. major* and *L. mexicana* showed that *ARG* is essential for *in vitro* growth in the absence of polyamines and needed for full infectivity in animal infections. The ARG protein is normally found within the parasite glycosome, and here we examined whether this localization is required for survival and infectivity. First, the localization of *L. amazonensis* ARG in the glycosome was confirmed in both the promastigote and amastigote stages. As in other species, *arg^−^ L. amazonensis* required putrescine for growth and presented an attenuated infectivity. Restoration of a wild type *ARG* to the *arg* ^−^ mutant restored ARG expression, growth and infectivity. In contrast, restoration of a cytosol-targeted ARG lacking the glycosomal SKL targeting sequence (*arg*ΔSKL) restored growth but failed to restore infectivity. Further study showed that the ARGΔSKL protein was found in the cytosol as expected, but at very low levels. Our results indicate that the proper compartmentalization of *L. amazonensis* arginase in the glycosome is important for enzyme activity and optimal infectivity. Our conjecture is that parasite arginase participates in a complex equilibrium that defines the fate of L-arginine and that its proper subcellular location may be essential for this physiological orchestration.

## Introduction

Leishmaniasis is the second most important infection caused by a protozoan and affects 12 million people worldwide (WHO). Considered to be one of the most neglected tropical diseases, leishmaniasis is endemic in almost all tropical and subtropical areas and causes a wide range of devastating and potentially deadly diseases. There is no vaccine to prevent leishmaniasis, and the drug arsenal to treat this disease presents many problems, including toxicity and increasing parasite resistance to common chemotherapies. Therefore, the study and validation of new drug targets are important for the development of effective chemotherapies.

The causative agents of leishmaniasis are protozoan parasites of the genus *Leishmania*, family Trypanosomatidae. The life cycle of *Leishmania* includes the extracellular promastigote form that resides in the midgut of the phlebotomine sand fly vector. The obligate intracellular amastigote form inhabits the phagolysosomes of mononuclear phagocytes (mainly macrophages) within the mammalian host. *Leishmania* is able to survive and replicate in these different environments by adapting to a wide range of temperatures, pH levels and nutrient availability and also by escaping the anti-proliferative defense molecules produced by the host cell [1], [2].

The amino acid L-arginine appears to play a key role in the survival of *Leishmania* in the mammalian host [3]–[5]. The modulation of the Th1/Th2 balance can induce either the death or proliferation of intracellular *Leishmania* in the macrophages [6]–[8], and L-arginine plays an important role in this process as a common substrate of the inducible nitric oxide synthase (iNOS) and the ARG of the host [9]. These enzymes are competitively regulated by type 1 (Th1) and type 2 (Th2) cytokines, with increased iNOS and decreased ARG levels contributing to parasite control in Th1 responses, and decreased iNOS and increased ARG levels contributing to parasite survival in Th2 responses. Host cell metabolism of L-arginine is further complicated by the fact that *Leishmania* parasites also express a highly active uptake pathway for arginine and their own ARG. *L. amazonensis* [10] and *L. mexicana* [11] ARG proteins and enzyme activities have been biochemically characterized, as have their coding genes. Characterization of mutant parasites lacking *ARG* (*arg* ^−^) in *L. mexicana* and *L. major* demonstrated that the *ARG* pathway is essential for *in vitro* proliferation of these parasites, rendering *arg* ^−^ parasites auxotrophic for polyamines [11], [12]. The *L. mexicana arg* ^−^ parasites showed attenuated infectivity in BALB/c mice, which was attributed to an increase in host nitric oxide (NO) production due to L-arginine availability [13]. In contrast, the *L. major arg* ^−^-attenuated infectivity in BALB/c mice was not due to NO overproduction. Moreover, the cytokine profile response induced by *L. major arg* ^−^ was not different from that induced by WT parasites, suggesting that the effect of ARG on *L. major* infection is not associated with the host immune response [14].

Previously, using *L. amazonensis* promastigotes expressing a glycosomal-targeted EGFP (enhanced green fluorescent protein), we showed that ARG is compartmentalized in glycosomes by co-localizing glycosomal EGFP with ARG immunolabeling [15]. Glycosomes are peroxisome-like organelles, unique to trypanosomatids, where a part of carbohydrate metabolism is compartmentalized [16], [17]. Another strategy revealed the same location for *L. mexicana* promastigote ARG using ARG fused to EGFP, which co-localized with a glycosomal marker [11]. These authors suggested that the glycosomal milieu is not essential for the role of ARG in polyamine biosynthesis [11]. The maintenance and importance of ARG glycosomal compartmentalization, however, was not further verified during parasite infection *in vitro* or *in vivo*. Here, after confirming that ARG remains in the glycosome during macrophage infection, we show that both the incorrect localization and the lack of ARG impair parasite proliferation and attenuate infection. The results of this study indicate that the proper sub-cellular compartmentalization of *L. amazonensis* ARG in the glycosome is important for enzyme activity and proper physiological functioning during parasite infection.

## Results

### *L. amazonensis* ARG remains in the glycosome in the amastigote form during macrophage infection

**Figure 1:**
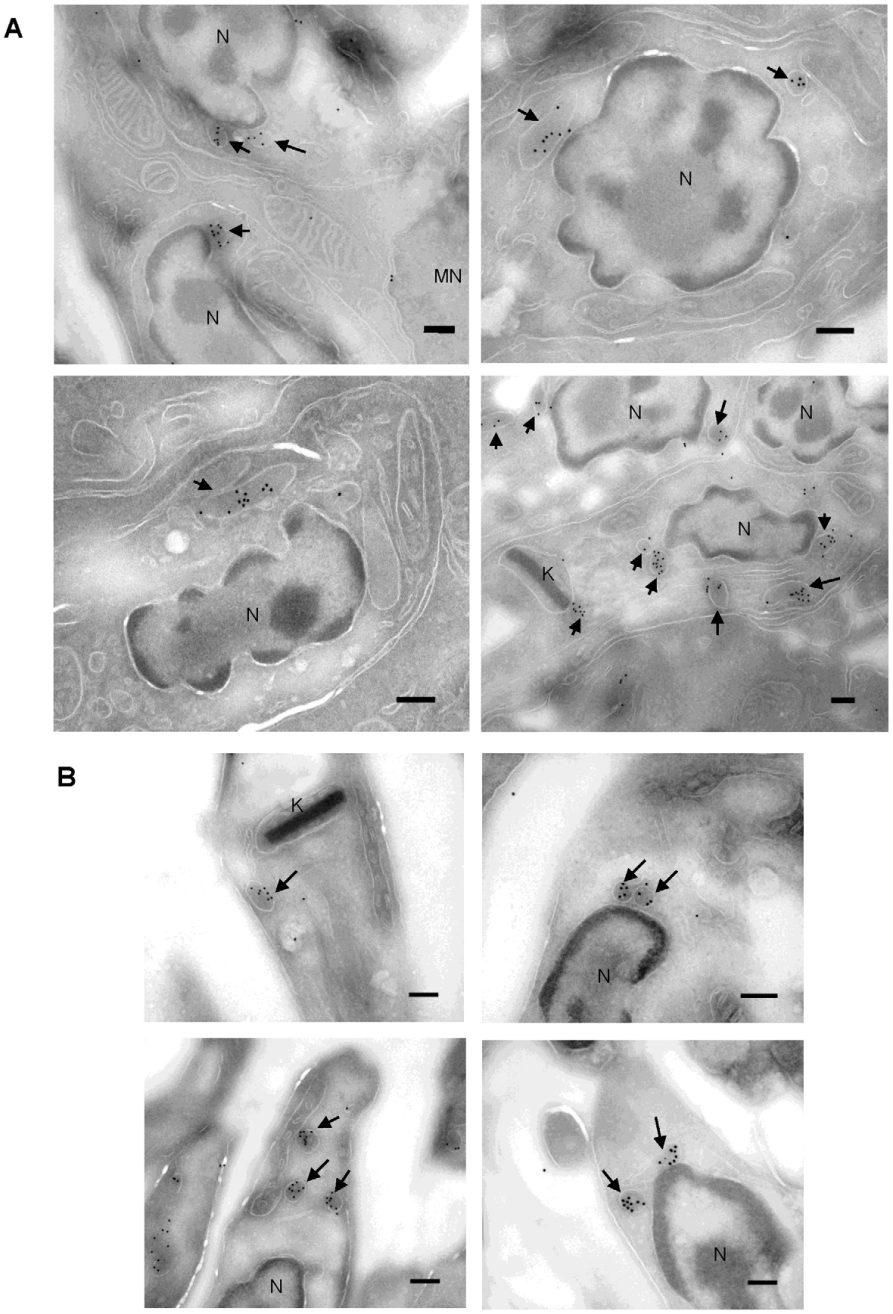
*L. amazonensis* ARG remains compartmentalized during macrophage *in vitro* infection. Cytological preparation probed for arginase immunolocalization via electron microscopy. (A) J774A 1 macrophages infected for 24 hours with *L. amazonensis* promastigotes in the stationary phase (4 different sections). (B) *L. amazonensis* promastigotes. Black arrows indicate compartmentalized ARG labeling. Parasite nuclei (N), macrophage nuclei (MN) and the kinetoplast (K) are indicated. Scale bar: 200 nm.

Infected J774A 1 macrophages were probed with anti-ARG polyclonal antibodies to determine ARG localization in the *L. amazonensis* amastigote form by electron microscopy (EM) immunolocalization experiments (Figure 1A). As a control, ARG immunolabeling was also performed with promastigotes (Figure 1B). These experiments revealed that the enzyme remains compartmentalized in a membrane-surrounded compartment, assumed to be the glycosome given its resemblance to the promastigote labeling pattern.

### Generation of an ARG-null mutant of *L. amazonensis* (arg^−^) and complemented lines bearing WT or a glycosomal targeting sequence deleted ARG (argΔSKL)

To study the role of *L. amazonensis* ARG during the parasite life cycle, an *ARG* null mutant (*arg* ^−^) was generated by homologous gene replacement (Figure S1A). As *Leishmania* do not undergo sexual recombination when grown *in vitro* [18], two successive rounds of targeting are required because *Leishmania* is disomic at most chromosomes [19]. The 5′ and 3′ untranslated coding regions (UTR) flanking the *ARG* open reading frame (ORF) were joined to fragments expressing resistance to Hygromycin B (*HYG*) or Puromycin (*PAC*) as described in the Methods Section. These targeting constructs were then introduced successively into WT *L. amazonensis*, ultimately yielding the homozygous knockout *ΔARG:HYG/ΔARG:PAC*, referred to hereafter as *arg* ^−^. Loss of *ARG* and generation of the planned replacements for both *ARG* alleles were confirmed by PCR (Figure S1B).

To restore *ARG* expression and to evaluate how its cellular location affects its physiological role in *L. amazonensis*, both WT and a modified *ARG* ORF lacking the C-terminal SKL glycosomal targeting sequence were stably integrated into the SSU rRNA locus of the *arg* ^−^ mutant, yielding lines referred as *arg* ^−^/+*ARG* and *arg* ^−^/+*arg*ΔSKL, respectively (Figure S2A). Successful introduction of the *ARG* ORF and integration into the SSU rRNA locus were confirmed by PCR analyses (Figure S2B).

### Characterization of arg^−^ and arg^−^/+ARG and arg^−^/+argΔSKL

**Figure 2:**
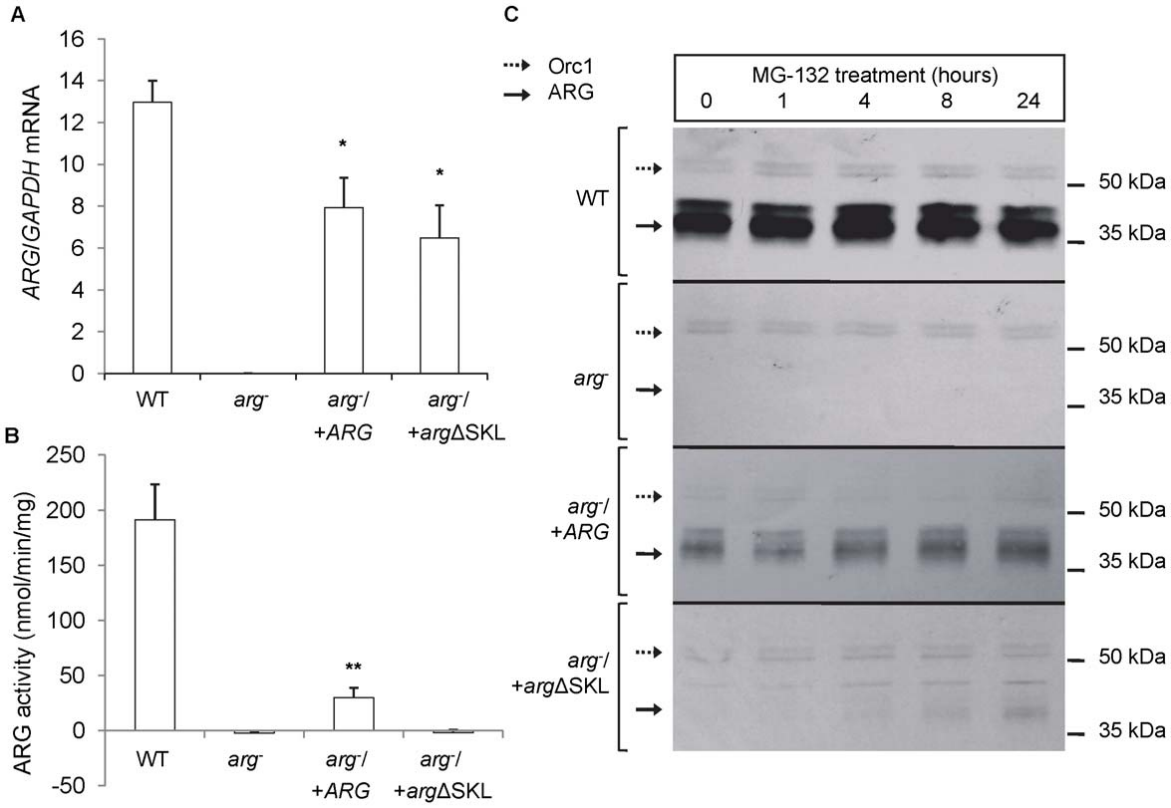
*L. amazonensis* ARG protein level and enzymatic activity are impaired by arginase mislocation. (A) *ARG* mRNA copies were determined by real time PCR and normalized by *GAPDH* mRNA copies for *L. amazonensis* wild type (WT), *ARG* knockout (*arg* ^−^), and the add-backs *arg* ^−^/+*ARG* and *arg* ^−^/+*arg*ΔSKL strains. The obtained values are the means (+/− SD) of at least 2 independent experiments each performed in duplicate. The values for *arg* ^−^/+*ARG* and *arg* ^−^/+*arg*ΔSKL are significantly different from those for *arg* ^−^ and WT (p<0.0015*, t test). (B) ARG enzymatic activity from protein extracts of the same strains was determined and is expressed as nmol/min/mg. The obtained values are the means (+/− SD) of 3 independent experiments each performed in triplicate. The values for *arg* ^−^/+*ARG* are significantly different from those for *arg* ^−^ and *arg* ^−^/+*arg*ΔSKL (p<0.0001**, t test). (C) ARG and Orc1 (loading control) levels were determined by western blotting of total cell lysates from the same strains before and after treatment with MG-132 50 µM for 24 hours.

The level of ARG mRNA expression was quantified by real-time PCR, normalized to the expression of GAPDH (Figure 2A). As expected, ARG mRNA expression was abolished in the arg- parasites. The add-back mutants arg−/+ARG and arg−/+argΔSKL showed substantial restoration of ARG mRNA expression (60% and 50%, respectively; Figure 2A). Treatment of WT and add-back parasites with Sinefungin and Actinomycin to completely inhibit mRNA synthesis [20] showed that the mRNA half-lives of the ARGs integrated into SSU rRNA locus are less than the WT ARG mRNA half-life (Text S1, Figure S3). Enzymatic assays similarly revealed that the *arg^−^* parasite lacked detectable ARG activity. Similar to the mRNA results, the *arg^−^*/*+ARG* add-back showed partial rescue of ARG activity (29.7 nmol/min/mg) to approximately 15% of the WT ARG activity (191.1 nmol/min/mg). In contrast, the *arg^−^*/+*arg*ΔSKL mutant did not show any detectable ARG activity (Figure 2B).

Western blot analysis using anti-ARG sera confirmed the complete absence of ARG in *arg* ^−^ and the lowered levels of ARG in the partially rescued *arg* ^−^/+*ARG* mutant parasites, to approximately 65% of the WT (Figure 2C and S4A; in these studies, reactivity with an anti-Orc1 antiserum was used as a loading control). Corroborating the ARG activity result, a weak labeling was observed in the *arg^−^*/+*arg*ΔSKL mutants.

**Figure 3:**
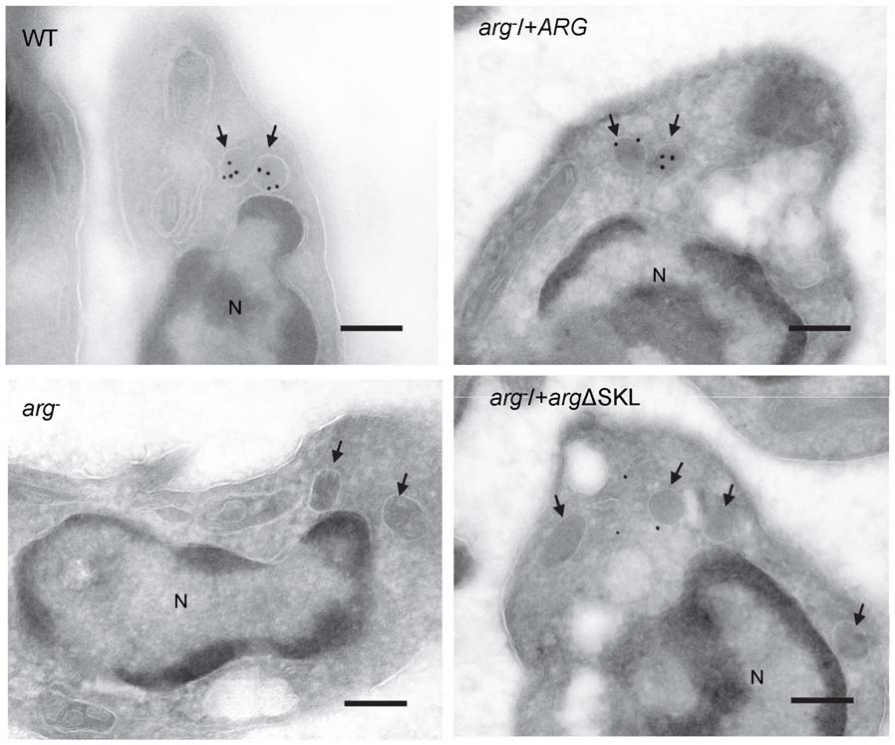
ARG cytosolic localization in *arg* ^−^/+*arg*ΔSKL add-back opposed to ARG glycosomal compartmentalization in WT and *arg* ^−^/+*ARG* add-back. Cytological preparations probed for ARG immunolocalization via electron microscopy of *L. amazonensis* wild type (WT), *ARG* knockout (*arg* ^−^), and the add-backs *arg* ^−^/+*ARG* and *arg* ^−^/+*arg*ΔSKL promastigotes. Images are representative of at least 8 different sections. Black arrows indicate membrane-surrounded organelles that present the same microscopic pattern as glycosomes. Parasite nuclei (N). Scale bar: 250 nm.

ARG immunolabeling by EM settled that the enzyme was compartmentalized in the glycosome in *arg* ^−^/+*ARG* parasites. Most importantly, EM study in *arg^−^*/+*arg*ΔSKL parasites showed that ARG was distributed throughout the cell cytosol, not compartmentalized, which confirms the incorrect targeting of ARG in *arg^−^*/+*arg*ΔSKL (Figure 3).

Seeking conditions that would allow the detection of argΔSKL, all parasites were treated with MG-132 (carbobenzoxy-L-leucyl-L-leucyl-leucinal), an inhibitor of proteasome activity. This approach resulted in a time-dependent ARG accumulation that enabled ARG detection in the *arg^−^*/+*arg*ΔSKL mutant. After 24 hours with MG-132, *arg^−^*/+*arg*ΔSKL labeling increased approximately 34%, which still corresponded to approximately 5% of the WT ARG levels submitted to the same treatment (Figure 2C and S4B). ARG accumulation in WT and *arg* ^−^/+*ARG* parasites was 40 and 45%, respectively, and the *arg* ^−^/+*ARG* ARG level was approximately 60% of the WT ARG level.

Treatment with MG-132 also enabled ARG to be localized in the add-back mutants by confocal immunofluorescence (Figure S5). These images confirmed the ARG accumulation after MG-132 treatment in WT and add-back mutants and also showed that ARG was not compartmentalized in *arg^−^*/+*arg*ΔSKL parasites, as was observed in the WT and *arg* ^−^/+*ARG* parasites, but was instead distributed throughout the cell cytosol, corroborating the incorrect targeting of ARG in *arg^−^*/+*arg*ΔSKL.

### L-arginine cellular concentration is modulated by ARG expression

L-arginine intracellular concentration was determined in WT and mutant parasites by HPLC analysis (Table 1). L-arginine levels in *arg* ^−^ parasites increased four-fold over WT (79±26 vs. 19±0.4 nmol/10^7^ parasites; p<0.05), while the level in the *arg* ^−^/+*ARG* add-back parasites was not significantly different from that in WT parasites (29±14 nmol/10^7^ parasites). In contrast, the L-arginine pool was significantly increased in the *arg* ^−^/+*arg*ΔSKL parasites (94±27 nmol/10^7^ parasites), comparable with that observed in the *arg* ^−^ parasites. The cellular concentration of other amino acids did not differ between WT parasites and any of the mutant parasites (data not shown). These results indicate that proper ARG localization and activity is important for the maintenance of L-arginine levels in *Leishmania*.

**Table 1:** L-arginine cellular concentration is increased in *arg* ^−^ and *arg* ^−^/+*arg*ΔSKL compared with WT and *arg* ^−^/+*ARG*.

	L-arginine cellular concentration (nmol/10^7^ parasites)
WT	19.07 (0.43)
*arg* ^−^	79.24 (25.86)*
*arg* ^−^/+*ARG*	28.57 (14.18)§
*arg* ^−^/+*arg*ΔSKL	93.57 (27.20)* #

L-arginine cellular concentration in *L. amazonensis* wild type (WT), *ARG* knockout (*arg* ^−^) and the add-backs *arg* ^−^/+*ARG* and *arg* ^−^/+*arg*ΔSKL, determined by HPLC analyses of cellular extracts from 10^7^ parasites. Values are the means (+/−SD) of a representative experiment performed in triplicate.

* indicates values significantly different compared with WT (p<0.05, t test).

§ and # indicates values with no significant difference compared with WT and *arg* ^−^, respectively.

### ARG activity is important for parasite growth, despite sub-cellular location

**Figure 4:**
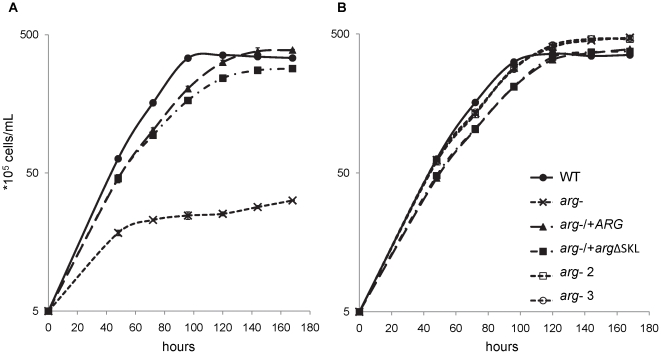
ARG absence impairs parasite growth *in vitro*. Growth curves of *L. amazonensis* wild type (WT), *ARG* knockout (*arg* ^−^) and the add-backs *arg* ^−^/+*ARG* and *arg* ^−^/+*arg*ΔSKL promastigotes in the absence (A) or presence (B) of 10 µM (*arg* ^−^ 2), 30 µM (*arg* ^−^ 3) or 50 µM (WT, *arg* ^−^, *arg* ^−^/+*ARG* and *arg* ^−^/+*arg*ΔSKL) putrescine. The obtained values are the means (+/− SD) of one representative experiment performed in triplicate.

To determine the importance of ARG levels and sub-cellular location for parasite proliferation, the growth of *arg* ^−^ and the genetically complemented parasites, with or without putrescine supplementation, were evaluated. These experiments were performed using M199 medium with reduced levels of fetal bovine serum (FBS, 0.5% rather than 10%) to minimize the effects arising from fetal calf serum-derived polyamines. The *arg* ^−^ parasites were not able to proliferate as well as WT parasites in medium without putrescine, reaching less than 1/10 of the WT stationary cell concentration (Figure 4A). As expected, putrescine medium supplementation with 10 to 50 µM led to equal growth rescue of *arg* ^−^ mutants (Figure 4B). Both the *arg* ^−^/+*ARG* and *arg* ^−^/+*arg*ΔSKL parasites grew similarly to WT parasites (Figure 4A). Thus, despite the impairment of ARG in the *arg* ^−^/+*arg*ΔSKL parasites, they were able to proliferate as well as WT and *arg* ^−^/+*ARG* parasites.

### ARG mislocation or absence impairs *L. amazonensis* infectivity

**Figure 5:**
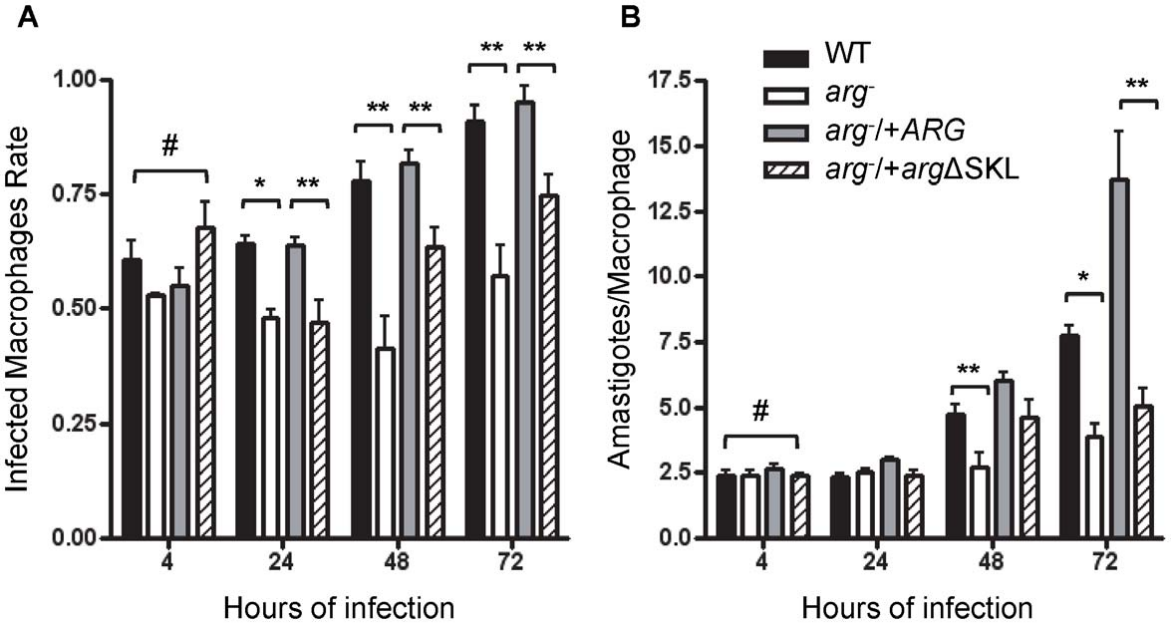
ARG absence or improper sub-cellular location impairs *L. amazonensis in vitro* infectivity. Peritoneal macrophages infected with *L. amazonensis* wild type (WT, white bars), *ARG* knockout (*arg* ^−^, black bars) and the add-backs *arg* ^−^/+*ARG* (grey bars) and *arg* ^−^/+*arg*ΔSKL (dashed bars) mutants for 48 and 72 hours. (A) Means (+/− SD) of the rate of macrophage infection from two experiments. (B) Means (+/− SD) of amastigotes per infected macrophage from two experiments. At least 150 macrophages were analyzed for each experiment. ^#^ indicates values with no significant difference (One-way ANOVA). * p<0.005 and ** p<0.05 (t test).

To determine the rates of macrophage infection 4, 24, 48 and 72 hours post-infection (Figure 5A) and the number of amastigotes per infected macrophage as an indicator of amastigote proliferation capacity (Figure 5B), the infectivity of WT and mutant parasites was first examined during *in vitro* infections of BALB/c peritoneal macrophages. All strains presented a similar behavior at 4 hours post-infection, indicating that the initial phagocytosis was not affected. After 24 hours post-infection, *arg* ^−^ parasites had a decreased macrophage infection rate compared with that of WT parasites. *arg* ^−^/+*arg*ΔSKL parasites showed a similar impairment, relative to WT, as the *arg* ^−^ parasites (Figure 5A, striped bars). As expected, *arg* ^−^/+*ARG* presented a WT-like macrophage infection rate and proliferation (Figure 5AB, gray bars). From 48 hours post-infection, a decreased amastigote proliferation was observed for *arg* ^−^ and *arg* ^−^/+*arg*ΔSKL parasites (Figure 5B, white and striped bars). From these data, the infectivity index (the rate of infected macrophages multiplied by the average number of amastigotes per infected cell) was calculated (Table 2). The infectivity index of *arg* ^−^ parasites was decreased by approximately 80% at 24 hours post-infection and 30% at 48 and 72 hours post-infection, compared with the WT parasite. The recovery in *arg* ^−^/+*ARG* parasite infectivity indexes confirmed that the observed decrease in the *arg* ^−^ infectivity index was due to the lack of ARG. Notably, *arg* ^−^/+*arg*ΔSKL parasites were unable to fully recover their infective capacity, presenting an infectivity index of approximately 75% at 24 and 48 hours post-infection and 50% at 72 hours, compared with WT parasites. These results indicate that ARG absence or incorrect localization impairs parasite *in vitro* infectivity.

**Table 2:** ARG absence or improper sub-cellular location impairs *L. amazonensis in vitro* infectivity.

Strain	4 h	24 h	48 h	72 h
WT	1.48 (0.27)	1.50 (0.23)	3.69 (0.70)	7.05 (0.64)
*arg^−^*	1.27 (0.11)	1.21 (0.05)	1.13 (0.64)*	2.20 (0.62)***
*arg^−^/+ARG*	1.46 (0.33)	1.92 (0.03)	4.94 (0.54)	13.03 (3.53)
*arg^−^/+argΔ*SKL	1.67 (0.25)	1.13 (0.30)**	2.91 (0.78)**	3.79 (1.20)**

Infectivity indexes (macrophage infection rate [Fig. 5A]×amastigotes/macrophage means [Fig. 5B]) of peritoneal macrophages infected with *L. amazonensis* wild type (WT), *ARG* knockout (*arg* ^−^) and the add-backs *arg* ^−^/+*ARG* and *arg* ^−^/+*arg*ΔSKL after 4, 24, 48 or 72 hours. The standard deviation is presented in brackets. Values are significantly different in relation to WT, with p-values: p≤0.01*, p = 0.007***; or in relation to *arg* ^−^/+*ARG*: p<0.03** (t test).

**Figure 6:**
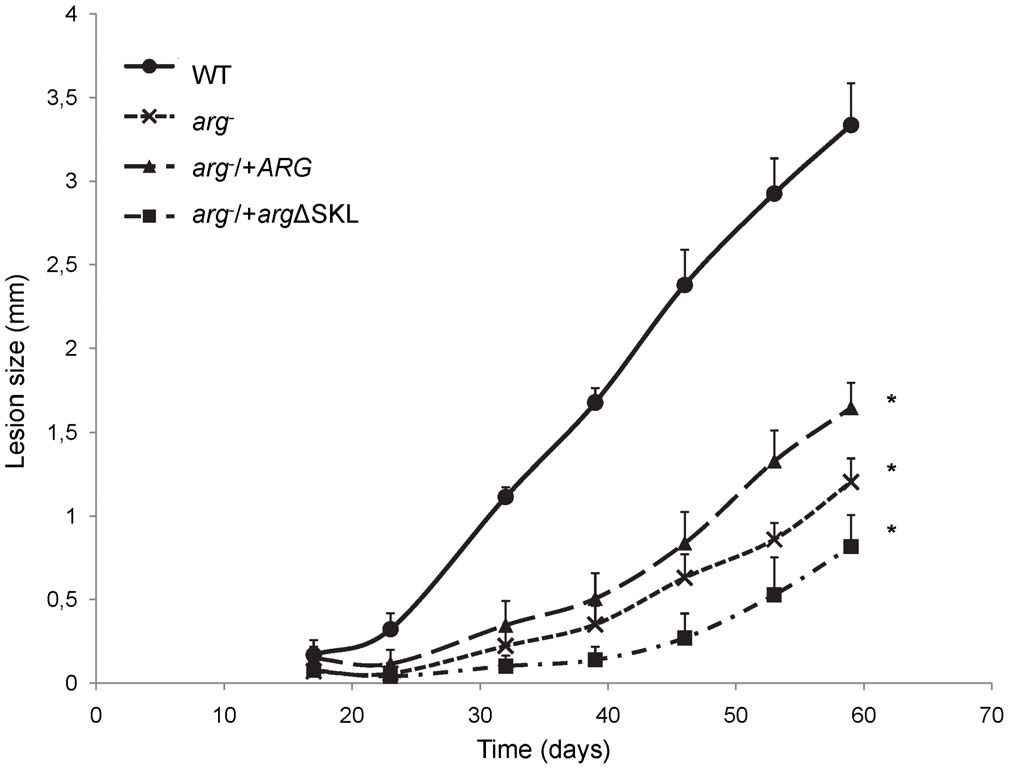
*L. amazonensis in vivo* infectivity is impaired by lack of ARG and incorrect location. BALB/c mice were infected in the posterior left footpad with 10^6^ stationary phase promastigotes cells of *L. amazonensis* wild type (WT), *ARG* knockout (*arg* ^−^) and the add-backs *arg* ^−^/+*ARG* and *arg* ^−^/+*arg*ΔSKL parasites, and lesion sizes were monitored weekly. Data are presented as the means (+/− SD) of 5 infected mice and are representative of at least 2 different strains of null and add-backs mutants.* p<0.005 (Two-way ANOVA).

The ability of the parasites to survive and induce lesions within susceptible BALB/c mice was then examined after footpad inoculation with 10^6^ stationary phase promastigotes (Figure 6). The *arg* ^−^ parasites induced a delayed lesion development compared with the WT parasites, consistent with the results of *arg* ^−^ mutants in other *Leishmania* species [13], [14]. After 59 days, the lesions induced by *arg* ^−^ parasites were approximately 36% smaller than those induced by WT parasites. Despite the substantial restoration of ARG enzyme levels, ARG metabolism, and full infectivity *in vitro*, the *arg* ^−^/+*ARG* parasites only partially rescued the progression of the infection. Notably, *arg* ^−^/+*arg*ΔSKL parasites showed a greater delay in lesion formation than observed with the *arg* ^−^ mutant. After 59 days, lesions induced by the *arg* ^−^/+*arg*ΔSKL parasites were approximately 76% smaller than those induced by the WT parasites and 32% smaller than those induced by the *arg* ^−^ parasites (p<0.0001, One-way ANOVA test). At this point, the WT-infected mice were sacrificed. After 16 weeks, the mice infected with the *arg* ^−^/+*arg*ΔSKL mutant presented lesions that corresponded to approximately 60% of the ones induced by the WT parasites (data not shown).

Taken together, these results show that restoring ARG expression in its native sub-cellular location at least partially recovers the infectivity of *L. amazonensis arg^−^* parasites. The presence of incorrectly located arginase (*arg* ^−^/+*arg*ΔSKL), however, did not improve the *arg^−^ in vitro* infectivity; instead, it resulted in an even more substantial loss in infectivity.

## Discussion

Previous studies have shown that *Leishmania* ARG is essential for polyamine synthesis [11] and enhances parasite infectivity and pathogenesis [13], [14]. ARG compartmentalization in the glycosome, however, has been considered unimportant for proper enzyme performance in *L. mexicana* physiology [11]. Here, we show the importance of ARG glycosomal compartmentalization in *L. amazonensis*, characterizing two add-back mutants: one with the native *ARG* ORF (*arg* ^−^/+*ARG*) and the other with a modified *ARG* ORF lacking the C-terminal SKL glycosomal targeting sequence (*arg* ^−^/+*arg*ΔSKL), which were generated from the initially obtained *L. amazonensis ARG* null mutant (*arg* ^−^) that did not express *ARG* mRNA or present ARG activity. *ARG* mRNA expression was only partially restored in both add-back mutants. Although the *ARG* add-back sequences were integrated into the non-native SSU rRNA locus, the partial rescue could be explained by the absence of the *ARG* 5′ and 3′ UTR native regulatory sequences. In fact, *ARG* mRNA half-life determination in both add-back mutants revealed a diminished stability when compared with WT.

The partial recovery in protein level and ARG enzymatic activity of the *arg* ^−^/+*ARG* add-back may result from the partial expression of *ARG* mRNA. Interestingly, despite the partial *ARG* mRNA expression recovery, the *arg* ^−^/+*arg*ΔSKL add-back showed faint ARG labeling in western blot assays and no ARG enzyme activity. At the EM level, however, ARG labeling confirmed the cytosolic location of the enzyme in these add-backs.

Considering that improperly located ARG could impair assembly of the trimer form of the active enzyme [15], we assumed that the mistargeted ARG could be being degraded via the proteasome pathway, which is widely known for its role in cellular quality control against misfolded proteins present in the cytosol [21]–[23]. Treatment with a trypanosome proteasome inhibitor, MG-132 [24], led to ARG accumulation in the WT and both add-backs parasites, enabling ARG detection in *arg* ^−^/+*arg*ΔSKL western blot and confirming the cytosolic location of ARG by confocal immunolabeling.

Regarding *in vitro* proliferation, the *arg* ^−^ null mutant is auxotrophic for polyamines, as previously shown for *L. mexicana* and *L. major* [11], [12]. Nevertheless, the *arg* ^−^/+*arg*ΔSKL axenic promastigotes were able to grow without putrescine supplementation to the same levels achieved by both WT and *arg* ^−^/+*ARG* parasites. We conjectured that the minimal amount of ARG detected via western blot and EM could lead to a level of ARG activity not detectable by the used dosage method but sufficient for the mutant to overcome the *in vitro* promastigote proliferation. There was, however, a significant impairment of *in vitro* and *in vivo* pathogenicity of the *arg* ^−^/+*arg*ΔSKL mutant. The *in vivo* infection results point to an important role for the *L. amazonensis* ARG in providing polyamines for amastigote proliferation in the host environment. In addition, incorrectly located ARG ablates parasite infectivity even more than its absence. This observation was confirmed by *in vitro* macrophage infection, which showed a reduced infectivity of the *arg* ^−^/+*arg*ΔSKL mutant at 24 hours post-infection. The initial steps of *in vitro* infection (4 hours post-infection) showed that the reduced infectivity was not due to the capacity of the mutants to enter the host cell. These infection data corroborate those reported for *L. mexicana* and *L. major arg* ^−^ mutants [13].

L-arginine availability is known to be important for the balance of the Th1/Th2 response in *Leishmania* infection [6], [25]. The four-fold increase in the L-arginine internal pool observed in *arg* ^−^ and *arg* ^−^/+*arg*ΔSKL mutants, associated with their impaired infectivity, could indicate that one of the roles of ARG during infection is modulating the availability of L-arginine, and consequently regulating the host immune response via the iNOS pathway [8], [9] or the MAPK pathway, which recently was indicated as another L-arginine-dependent pathway [26]. In addition, modulating L-arginine availability would also affect parasite infectivity by influencing the NO production of the parasite [27].

Another participant in this complex balance is the host ARG type I, which is also an important participant during parasite pathology and would be affected by the parasite's modulation of L-arginine availability [28]. Indeed, further studies using the mutant parasites described in this work demonstrated that the internal pool of L-arginine is important in uptake regulation by modulating the amino acid transporter expression. We previously showed that the expression of the arginine transporter is modulated by arginase activity [29].

To summarize, our results indicate the importance of parasite ARG glycosomal localization for its role in the complex equilibrium that defines L-arginine fate and optimizes parasite infectivity. We speculate that the glycosome contains the optimal L-arginine concentration for arginase optimal activity. Our results differ from those of previous studies with *L. mexicana*, implying that the ARG sub-cellular milieu is not essential for its role in polyamine biosynthesis [11]. Other studies, however, have shown that the compartmentalization of certain enzymes in the glycosome is essential, and even minor mislocations of glycosomal enzymes, such as phosphoglycerate kinase (PGK), are lethal in the bloodstream form of *Trypanosome brucei* [30]–[32]. In addition, it the compartmentalization of glycolytic enzymes has long been known to improve glycolysis energy production in trypanosomatids by circumventing the low energy production (from the conversion of glucose to pyruvate) that occurs during the mammal cycle phase [33], [34]. Additionally, glycolysis compartmentalization in the glycosomes prevents the accumulation of toxic intermediates generated during glucose degradation [35]. A recent study showed that glycosomal localization of *Leishmania major* dihydroxyacetonephosphate acyltransferase is important for lipophosphoglycan synthesis [36].

Together with previous studies, our work indicates that both parasite and host ARGs are important for *Leishmania* infectivity. Because of the high similarity between these two enzymes, however, the screening of specific molecular inhibitors to target the parasite ARG has not been so promising regarding the pursuit of new leishmaniasis chemotherapies [37], [38]. Considering the location of ARG and its importance in modulating L-arginine availability, we propose that studies regarding *Leishmania* protein sorting mechanisms and L-arginine transport to the parasite glycosome are fundamental for understanding parasite physiology and for the screening of new drugs that would block protein glycosome import or substrate transport to the parasite glycosome, thereby compromising parasite survival in the host. This approach should be promising in the pursuit of new leishmaniasis chemotherapies.

## Materials and Methods

### Cell cultures

*Leishmania amazonensis* (MHOM/BR/1973/M2269) promastigotes were maintained in culture at 25°C in M199 (Invitrogen, Grand Island, NY, USA) supplemented with 10% (v/v) heat-inactivated fetal bovine serum (FBS; Invitrogen) or 0.5% FBS for growth experiments. J774A 1 macrophages were cultured at 37°C in 5% CO_2_ in RPMI medium (Invitrogen) supplemented with 10% (v/v) heat-inactivated FBS.

### Molecular constructs for the generation of arg^−^ null and genetically supplemented mutants

For targeted gene replacement of *L. amazonensis ARG*, the 5′ UTR-coding *ARG* flanking region was obtained by PCR using oligonucleotides 1 and 2 (Table S1) and genomic DNA from *L. amazonensis* as a template. The amplicon was cloned in pGEM-T easy (Promega, Madison, WI, USA) and purified after digestion with *Eco*RI and *Kpn*I. The fragment was inserted into a similarly digested pUC19 vector DNA. The 3′ UTR-coding *ARG* flanking region was obtained by PCR using oligonucleotides 3 and 4 (Table S1) and genomic DNA from *L. amazonensis* as a template. The amplicon was cloned in pGEM-T easy (Promega) and purified after digestion with *Xba*I and *Pst*I. The fragment was inserted into the similarly digested 5′ UTR-coding *ARG* containing pUC19 plasmid, yielding an intermediate, p5A3A, into which drug markers could be inserted between the 5′ and 3′ UTR coding *ARG* flanking regions.

The fragment containing the *HYG* ORF plus the 5′ *DHFR* region, encompassing expression regulating elements, was obtained by PCR using oligonucleotides 5 and 6 (Table S1) and pXGHYG DNA as a template. The amplicon was cloned into pGEM-T easy (Promega). The recombinant plasmid was purified and digested with *Kpn*I and *Xba*I. The produced fragment was inserted into the intermediate p5A3A, yielding the p5AHYG3A construct (Fig. S1A). The same procedure was performed for the *PAC* ORF using the pXGPAC DNA template, which yielded the p5APAC3A construct (Fig. S1A).

Gene-targeting linear fragments, which were to be used in transfection experiments, were obtained by PCR amplification using p5AHYG3A or p5APAC3A plasmid DNA as templates with oligonucleotides 7 and 8 (Table S1), which flank the region containing the *HYG/PAC* cassette between the 5′ and 3′ *ARG*. For replacement of the first *ARG* allele, 8 µg of purified fragment containing the *HYG* cassette was transfected into *L. amazonensis* using the high-voltage electroporation protocol described elsewhere [39]. The transfectants were plated on semisolid medium containing 30 µg/mL hygromycin B (Invitrogen). Several clones were obtained, and the correct insertion was verified by PCR analyses (Fig. S1B).

Several of the heterozygous replacements (*ARG*/*Δarg::HYG*) were subjected to a second transfection round with 8 µg of the purified fragment containing the *PAC* cassette for replacement of the second *ARG* allele. The transfectants were plated on semisolid medium containing 30 µg/mL hygromycin B and 30 µg/mL puromycin (Sigma, St. Louis, MO, USA), as well as 50 µM putrescine (Sigma) to circumvent auxotrophy [12]. Numerous clonal lines were obtained, and successful replacements of *ARG* alleles were certified by PCR amplification analysis (Fig. S1B). These *Δarg::HYG/Δarg::PAC ARG* knockout lines are referred to in the text as *arg* ^−^.

For genetic complementation of *arg* ^−^ null parasites, the *ARG* ORF with (*ARG*) and without the SKL glycosomal targeting signal (*arg*ΔSKL) were obtained by PCR amplification from *L. amazonensis* genomic DNA using the oligonucleotide pairs 9/10 or 9/11 (Table S1), respectively. The amplicons were cloned into pGEM-T easy (Promega). Plasmid DNA was digested with *Bam*HI, and the purified fragments were inserted into a similarly digested pIR1PHLEO vector [40], yielding the pIR1_*ARG* and pIR1_*arg*ΔSKL constructs, respectively (Fig. S2A).

To restore ARG expression in *arg* ^−^ parasites, linear fragments targeting for SSU rRNA insertion were obtained by digesting pIR1_*ARG* and pIR1_*arg*ΔSKL with *Swa*I, followed by dephosphorylation. A total of 8 µg of the purified fragments was transfected into *arg* ^−^ parasites using the same electroporation conditions as described above. The transfectants were plated on semisolid medium containing 30 µg/mL hygromycin B, 30 µg/mL puromycin, 50 µM putrescine and 20 µg/mL phleomycin (Sigma). Several clonal lines were obtained, and correct insertion was confirmed by PCR amplification analyses (Fig. S2B). These add-back lines are referred to in the text as *arg* ^−^/+*ARG* or *arg* ^−^/+*arg*ΔSKL, respectively.

### Reverse transcription and quantitative real-time PCR

RNA was obtained using TRIzol® Reagent (Invitrogen) or RNeasy® Micro kit (Qiagen, Germantown, MD), following the manufacturers' instructions. cDNA was synthesized using 200 U RevertAid™ Reverse Transcriptase (Fermentas Life Sciences, Burlington, Ontario, Canada) with 20 nmol oligo dT or 10 µM Random Hexamers (Applied Biosystems, Carlsbad, CA, USA) and 1 µg total RNA in a final volume of 13 µL. The RNA was denatured at 70°C for 5 minutes and then cooled to 15°C; next, 4 µL of 5× Buffer and 2 µL of 10 mM dNTP were added. The reaction was heated to 37°C for 5 minutes, and then 1 µL (200 U) of Reverse Transcriptase was added to the reaction, followed by incubation at 42°C for 60 minutes. The enzyme was inactivated at 75°C for 15 minutes, and the reaction was stored at −20°C. A negative control containing all reaction components except the enzyme was included and submitted to real-time PCR to exclude the possibility of DNA contamination in the RNA samples.

For real-time PCR of the cDNA synthesized from total RNA extracted with TRIzol® Reagent (Invitrogen), one-tenth of the reverse transcription product was used as a template. For real-time PCR of cDNA synthesized from total RNA extracted with RNeasy® Micro kit (Qiagen), a one-thousandth dilution was used as a template for mRNA quantification, or a one-hundred-thousandth dilution was used for SSU rRNA quantification. The reactions were performed with 0.2 µM of each corresponding primer pair and SYBR® Green (Applied Biosystems) in a final volume of 50 µL. For *L. amazonensis ARG* and *GAPDH* quantification, the specific primers 21/22 and 23/24, respectively, were used (Table S1); the PCR reaction consisted of 40 cycles of 30 seconds at 94°C, followed by 30 seconds at 61°C. For *L. amazonensis* SSU rRNA quantification, the specific primers 25/26 were used (Table S1); the PCR reaction consisted of 50 cycles of 20 seconds at 94°C, followed by 50 seconds at 62°C. Quantification of target gene expression was performed according to a standard curve prepared from a ten-fold serial dilution of a quantified and linearized plasmid containing the DNA segment to be amplified.

### Arginase activity assay

ARG activity was assayed by measuring urea production using the QuantiChrom™ Urea Assay Kit (DIUR-500) (BioAssay Systems, Hayward, CA). *Leishmania* promastigote pellets (5–8×10^7^ cells) were washed twice with PBS and resuspended in 40 µL of 150 mM Tris-HCl pH 7.8 buffer containing 1 mM MnSO_4_ and protease inhibitors (50 mg/mL ITS, 1 mM fenantroline, 2 mM benzamidine, 1 mM phenylmethanesulfonylfluoride [PMSF] and 2.5% [v/v] protease inhibitor cocktail [Sigma]). The cells were lysed by thermal shock (10 cycles of alternating freezing in liquid nitrogen and defrosting in a 42°C water bath). The *Leishmania* total protein extract obtained was clarified by centrifugation at 10000×g for 10 minutes at 4°C prior to the quantification of protein content by Bio-Rad Protein Assay (Bio-Rad Laboratories, Hercules, CA). A total of 10 µg of protein extract was used for L-arginine hydrolysis initiated by the addition of 50 mM glycine and 80 mM L-arginine (pH 9.6) in a final volume of 100 µL. Incubation was performed at 37°C, and the reaction was stopped by transferring 5 µL of the previous reaction to 200 µL of the QuantiChrom™ Urea Assay Kit working reagent. The samples were incubated for 2 hours at room temperature in the dark, and the urea formed was colorimetrically quantified at 450 nm.

### Anti-*L. amazonensis* ARG polyclonal serum production

Purified recombinant arginase from *L. amazonensis* was obtained as described previously [15]. Briefly, *E. coli* BL21(DE3)pLysS cells containing pRSET-*ARG* were induced for recombinant ARG expression with 1 mM IPTG at 37°C for 3 hours in the presence of 10 mM MnSO_4_. Recombinant arginase was obtained by submitting protein lysates of these cells to a purification process consisting of immobilized metal affinity chromatography followed by ionic exchange chromatography.

A New Zealand white rabbit was immunized by subcutaneous injection with 2 mg of the purified *L. amazonensis* ARG for the production of a polyclonal antiserum against *L. amazonensis* ARG.

### Western blotting

Parasites treated with or without 50 µM MG-132 (Z-Leu-Leu-Leu-al, Sigma), an inhibitor of proteasome activity, were collected and suspended in 1× Laemmli loading buffer. Samples (5×10^6^ cells/lane) were boiled for 5 minutes, subjected to SDS/PAGE and transferred to nitrocellulose membranes (Hybond-C, Amersham Biosciences, Little Chalfont, Buckinghamshire, England). The membranes were blocked with 5% non-fat dry milk in PBS containing 0.1% of Tween-20 for 1 hour. Protein loading was assessed with a rabbit anti-Orc1 polyclonal antibody [41] at a dilution of 1∶500. A rabbit anti-*L. amazonensis* ARG polyclonal antibody was used at a dilution of 1∶250. A peroxidase-conjugated goat anti-rabbit IgG (Santa Cruz Biotechnology, Santa Cruz, CA, USA) antibody was used at a dilution of 1∶1000. Labeling was revealed with hydrogen peroxide and 4-chloro-naphthol. Labeling densitometry was performed using NIH ImageJ 1.44 software.

### Confocal Immunofluorescence

WT and mutant cells were fixed in PBS/paraformaldehyde 4% for 1 hour at room temperature. Fixed cells were spotted on microscope slides and allowed to air dry for slide attachment. Samples were subjected to a 5-minute treatment with PBS/Triton 1% for cell permeabilization, followed by blocking with PBS/BSA 1% for 1 hour. Then, the samples were sequentially incubated with a rabbit anti-arginase antibody at a dilution of 1∶2000 for 18 hours at 37°C and an anti-rabbit IgG Fitc-conjugated antibody (Sigma) at a dilution of 1∶800 for 2 hours at 37°C in the presence of 3 µM DAPI (4′,6′-diamidino-2-phenylindole, Invitrogen), for DNA-rich structure staining. All steps were followed by several washes with PBS. The slides were mounted in Vectashield (Vector, Burlingame, CA) to reduce bleaching. Samples were examined with a Zeiss LSM 510 confocal laser microscope using Argon and Enterprise excitation lasers (Carl Zeiss, Oberkochen, Germany).

### Actinomycin and Sinefungin treatments

RNA half-life was determined using 10 µg/mL Actinomycin D (Sigma) and 2 µg/mL Sinefungin (Sigma) as described previously [20]. At different times, the parasites were collected, and the treatments were stopped by the addition of RNeasy® Micro kit RLT Lysis Buffer (Qiagen) for RNA extraction, cDNA synthesis and real-time PCR analyses as previously described. Data regression for one-phase exponential decay was performed using GraphPad Prism 4.0 software (San Diego, CA, USA).

### Amino acids cellular concentration HPLC analyses

Parasites (1×10^7^) were frozen, homogenized with 80% ethanol (Merck KGaA, Darmstadt, Germany) and concentrated in a speed vac (45°C, 1 hour). The samples were resuspended in 2 mL of water and centrifuged at 20,000×g for 2 minutes. The supernatant was passed through a mini-column Sep-Pak C_18_ (Waters), previously conditioned with 35% methanol (v/v), and sequentially eluted with 65% methanol (v/v). Aliquots of 100 µL were derivatizated with 100 µL of OPA derivatization solution (1 mL of 0.3 M OPA in methanol, 50 µL of mercaptoethanol and 10 mL of 0.1 M borate buffer, pH 9.5) during the 10 seconds before injection. Amino acids in each sample were analyzed by high-performance chromatography (HPLC) on a C_18_ column (Shimadzu Shim-pack CLC ODS). Solvent 1 (65% methanol; Merck) and solvent 2 (50 mM sodium acetate, 50 mM sodium phosphate, 1 mL acetic acid, 2% methanol and 2% tetrahydrofuran, pH 7.5), used in the mobile phase, were combined in the following gradient based on the percent of solvent 2 (35–40% [0.01–18 min], 40–65% [18–24 min], 65–68% [24–35 min] and 68–100% [35–45 min]) at a flow rate of 1 mL/min. Fluorescence excitation at 250 nm and emission at 480 nm were used for amino acid detection. The retention times and the areas of the peaks were measured by comparison with known quantities of standards amino acids: alanine, aspartic acid, glutamic acid, asparagine, serine, arginine, glutamine, histidine, aminobutiric acid, glycine, threonine, alanine, tyrosine, methionine, tryptophan, valine, phenylalanine, isoleucine, leucine, lysine and ornithine.

### In vitro macrophage infections

Peritoneal murine macrophages were obtained from BALB/c mice. The animals were sacrificed in a CO_2_ chamber, following the recommendations of the Animal Experimentation Ethical Committee. A total of 5 mL of PBS was injected into the peritoneal cavity. The peritoneum was rocked gently several times, and the buffer was aspirated. The aspirated cell suspension was washed with PBS at 1500×g for 10 minutes at 4°C, and then the cells were counted and resuspended in RPMI 1640 medium (Invitrogen) supplemented with 10% (v/v) heat-inactivated FBS (Invitrogen). Cells (2×10^5^) were seeded into each chamber of an 8-well glass chamber slide (Lab-Tek Chamber Slide; Nunc, Naperville, IL) and incubated for 18 hours at 34°C in 5% CO_2_. Non-adherent cells were washed away with fresh medium, and 10^6^ stationary phase promastigotes were added to each well. After 4 hours of incubation at 34°C in 5% CO_2_ non-phagocytized promastigotes were washed away with fresh medium. A sample was fixed and stained with Giemsa (Merck KGaA, Darmstadt, Germany). The remaining infected macrophages were incubated for an additional 20, 44 or 68 hours. After each period, the samples were fixed and stained as above. The infectivity indexes (rate of infected macrophages multiplied by the average number of amastigotes per macrophage) were calculated by randomly counting at least 150 macrophages per slide.

### Mouse infection

The virulence of WT and mutant *L. amazonensis* was evaluated after footpad inoculation of BALB/c mice. Groups were injected subcutaneously into the posterior left footpad with 10^6^ stationary phase promastigotes. Infections were monitored weekly by subtracting the thickness (analyzed with a caliper) of the injected footpad from that of the control (right) footpad.

### Electron Microscopy

The electron immunolocalization experiments were performed at the Molecular Microbiology Imaging Facility of Washington University School of Medicine (St. Louis, MO).

J774A 1 macrophages were infected *in vitro* as described above for peritoneal macrophages. *L. amazonensis* promastigotes or the infected macrophages were fixed in 4% paraformaldehyde/0.05% glutaraldehyde (Polysciences Inc., Warrington, PA) in 100 mM PIPES/0.5 mM MgCl_2_ (pH 7.2) for 1 hour at 4°C. The samples were embedded in 10% gelatin and infiltrated with 2.3 M sucrose/20% polyvinyl pyrrolidone in PIPES/MgCl_2_ overnight at 4°C. The samples were trimmed, frozen in liquid nitrogen and sectioned with a Leica Ultracut UCT cryo-ultramicrotome (Leica Microsystems Inc., Bannockburn, IL). Anti-ARG polyclonal rabbit serum was used as a probe and visualized using an 18-nm colloidal gold-conjugated goat anti-rabbit antibody (Jackson ImmunoResearch Laboratories, Inc., West Grove, PA). The sections were washed in PIPES buffer, followed by a water rinse, and stained with 0.3% uranyl acetate/2% methyl cellulose. The samples were examined with a JEOL 1200EX transmission electron microscope (JEOL USA Inc., Peabody, MA). All labeling experiments were conducted in parallel with controls in which the primary antibody was omitted. These controls were consistently negative.

### Statistical analysis

Statistical significance was determined by Student's t test or ANOVA test using Graph Pad Prism Software (GraphPad Software, Inc., La Jolla, CA, USA). The obtained p-values are indicated throughout the results.

## Supporting Information

Text S1 ***mRNA half-lives of both ARGs integrated into SSU rRNA locus is less than WT.***
(DOC)

Figure S1 **Generation of** ***L. amazonensis ARG*** **-null mutant (** ***arg*** **^−^).** (A) Gene replacement strategy. The 5AHYG3A/5APAC3A targeting fragments are shown above the *ARG* chromosomal locus. The arrows indicate the position of oligonucleotides (Table S1) used for PCR confirmation of planned insertions. The expected amplicons and their size in base pairs are indicated by brackets. (B) Agarose gels of the obtained amplicons from *L. amazonensis* wild type (WT), *ARG* heterozygous (+/−) and two *ARG* knockout clone (*arg* ^−^) genomic DNA templates with the indicated pairs of primers.
(TIFF)

Figure S2 **Generation of** ***L. amazonensis*** **complemented lines bearing WT or a glycosomal targeting sequence deleted** ***ARG*** **(** ***arg*** **ΔSKL).** (A) Insertion strategy into SSU rRNA locus. The targeting fragments containing *ARG* or *arg*ΔSKL (without SKL), selection marker *Phleo* and flanking recombination sequences are shown above the chromosomal SSU rRNA locus. The arrows indicate the position of oligonucleotides (Table S1) used for PCR confirmation of planned insertions. The expected amplicons and their size in base pairs are indicated by brackets. (B) Agarose gel showing the obtained amplicons from *L. amazonensis* wild type (WT), *ARG* knockout (*arg* ^−^) and the add-backs *arg* ^−^/+*ARG* and *arg* ^−^/+*arg*ΔSKL mutants genomic DNA templates with the indicated pairs of primers.
(TIFF)

Figure S3 **Half-lives of** ***arg*** **^−^/+** ***ARG*** **and** ***arg*** **^−^/+** ***arg*** **ΔSKL add-back parasites** ***ARG*** **mRNA are shorter than WT** ***ARG*** **mRNA.** Relative copy-numbers of *ARG* (A) and *GAPDH* (B) mRNA, normalized by SSU rRNA expression, of *L. amazonensis* wild type (WT), *ARG* knockout (*arg* ^−^), and the add-backs *arg* ^−^/+*ARG* and *arg* ^−^/+*arg*ΔSKL treated with actinomycin/sinefungin for mRNA decay determination. The obtained values are the means (+/− SD) of 3 independent experiments in duplicate. The black lines represent the exponential decay fitting with R^2^>0.98. t_1/2_ indicates the half-lives determined for each decay curve.
(TIFF)

Figure S4 **Proteasome inhibition with MG-132 causes ARG accumulation.** ARG and Orc1 (loading control) levels in *L. amazonensis* wild type (WT, white bars), and the add-backs *arg* ^−^/+*ARG* (gray bars) and *arg* ^−^/+*arg*ΔSKL (dashed bars) were determined by western blot after 0 to 24 hours of treatment with MG-132 50 µM (Fig. 1C). (A) ARG quantification before MG-132 treatment. Values were normalized by Orc-1 quantification and are the means (+/− SD) of duplicates of a representative experiment. (B) The increase in ARG/Orc-1 levels after 1, 4, 8 and 24 hours of treatment with MG-132. ARG/Orc-1 values for each strain were normalized by ARG/Orc-1 level of the same strain before treatment.
(TIFF)

Figure S5 **Inhibition of proteasome activity leads to ARG accumulation confirming cytosolic location in** ***arg*** **^−^/+** ***arg*** **ΔSKL cells.** *L. amazonensis* wild type (WT), *ARG* knockout (*arg* ^−^), and the add-backs *arg* ^−^/+*ARG* and *arg* ^−^/+*arg*ΔSKL parasites were treated (+) or not (−) with 50 µM MG-132 during 24 hours and then submitted to immunofluorescence assays to determine ARG sub-cellular localization. (A) Blue labeling, nuclei staining with DAPI. (B) Green labeling, ARG immunolabeling. (C) Phase contrast. (D) Merge of A, B and C. Images are representative of at least 5 different fields. Scale bar: 2 µm.
(TIFF)

Table S1 **Nucleotide sequences of primers used.**
(DOCX)
