# Supplementary material for: Leishmania amazonensis Arginase Compartmentalization in the Glycosome Is Important for Parasite Infectivity
Source: PLoS One. 2012 Mar 30;7(3):e34022. doi: 10.1371/journal.pone.0034022 (PMC3316525; doi:10.1371/journal.pone.0034022)
Supplement: Text S1 — mRNA half-lives of both ARGs integrated into SSU rRNA locus is less than WT. (DOC) [file pone.0034022.s001.doc]

**Supporting Information**

*mRNA half-lives of both ARGs integrated into SSU rRNA locus is less than WT*

To evaluate RNA turnover and determine mutant *ARG* mRNA half-lives, we treated WT and add-back parasites with Sinefungin and Actinomycin to completely inhibit mRNA synthesis [18]. Following treatment for up to 90 min, *ARG* and *GAPDH* mRNA levels were measured and normalized to SSU rRNA levels (Fig. S3). As expected, *GAPDH* showed a similar turnover in all lines. While WT *ARG* mRNA was extraordinarily stable (t_1/2_ nearly 500 min), both the *ARG* and *arg*ΔSKL add-back mRNAs were more unstable, with a t_1/2_ of 138 or 33 min respectively. This difference may reflect the fact that the *ARG* mRNAs bear flanking sequences from the pIR vectors for expression rather than the normal ARG flanking sequences.
