# Supplementary figures and images for: Leishmania amazonensis Arginase Compartmentalization in the Glycosome Is Important for Parasite Infectivity
Source: PLoS One. 2012 Mar 30;7(3):e34022. doi: 10.1371/journal.pone.0034022 (PMC3316525; doi:10.1371/journal.pone.0034022)

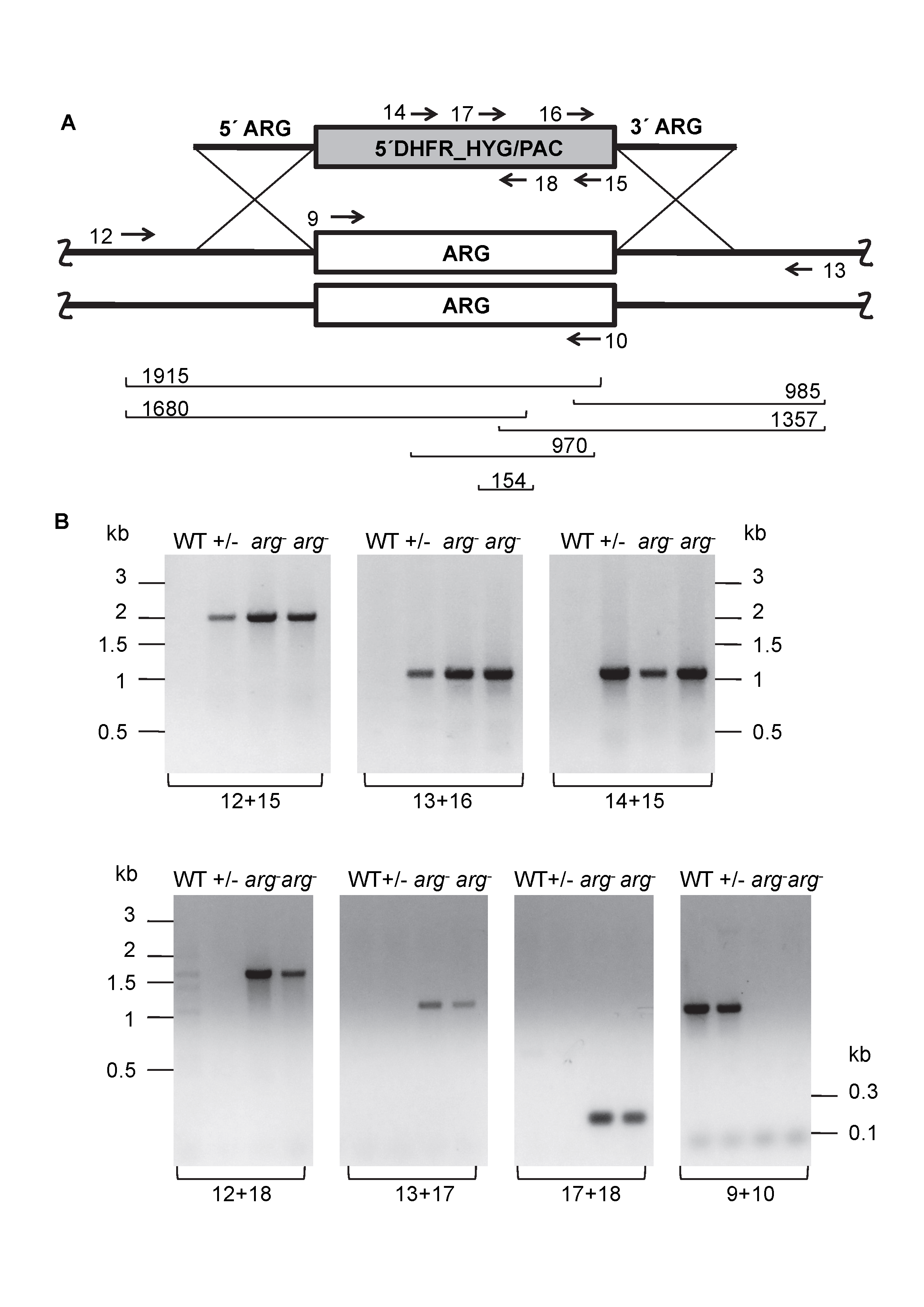

Supplement: Figure S1 — Generation of L. amazonensis ARG -null mutant ( arg −). (A) Gene replacement strategy. The 5AHYG3A/5APAC3A targeting fragments are shown above the ARG chromosomal locus. The arrows indicate the position of oligonucleotides (Table S1) used for PCR confirmation of planned insertions. The expected amplicons and their size in base pairs are indicated by brackets. (B) Agarose gels of the obtained amplicons from L. amazonensis wild type (WT), ARG heterozygous (+/−) and two ARG knockout clone (arg −) genomic DNA templates with the indicated pairs of primers. (TIFF) [file pone.0034022.s002.tiff]

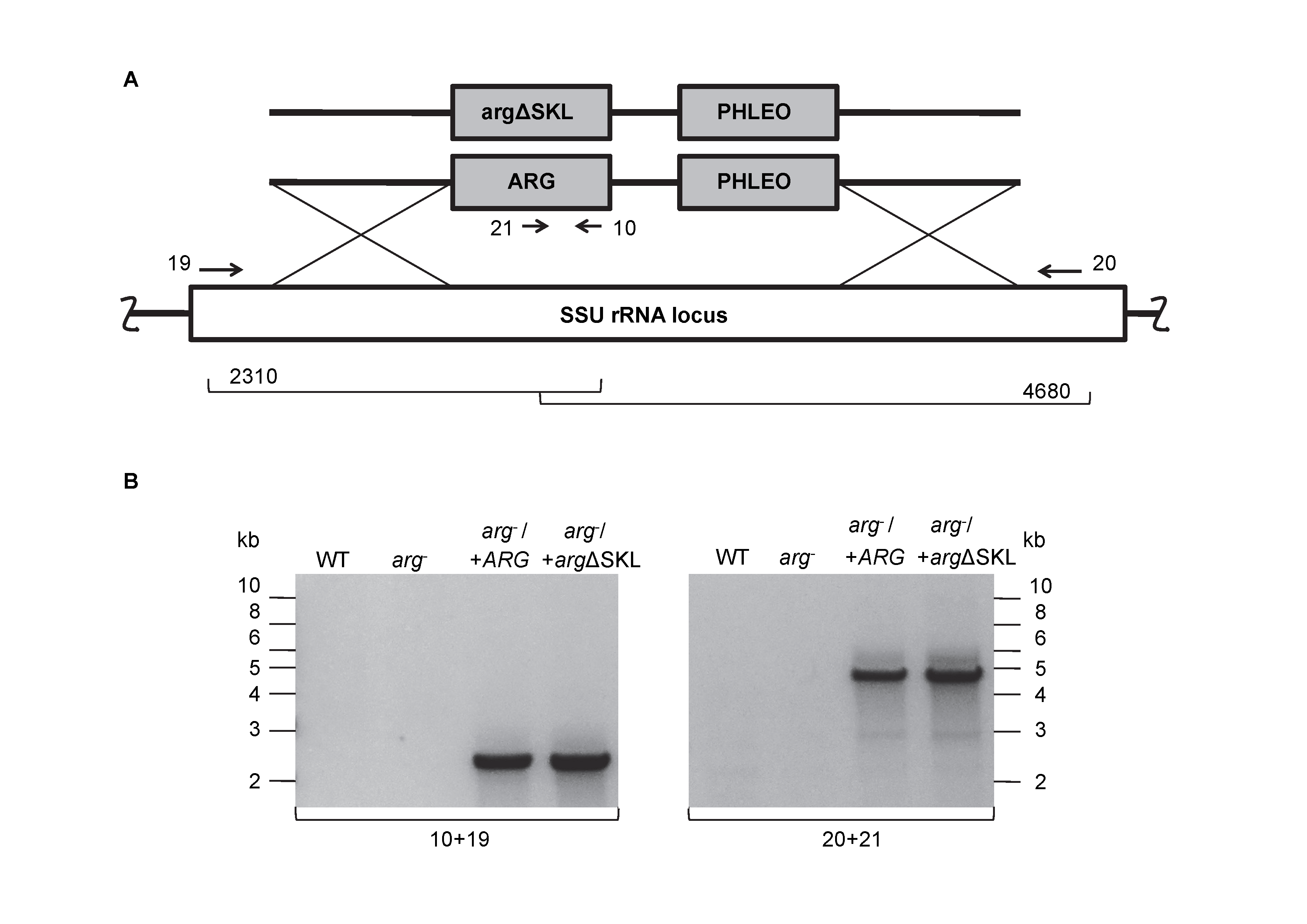

Supplement: Figure S2 — Generation of L. amazonensis complemented lines bearing WT or a glycosomal targeting sequence deleted ARG ( arg ΔSKL). (A) Insertion strategy into SSU rRNA locus. The targeting fragments containing ARG or argΔSKL (without SKL), selection marker Phleo and flanking recombination sequences are shown above the chromosomal SSU rRNA locus. The arrows indicate the position of oligonucleotides (Table S1) used for PCR confirmation of planned insertions. The expected amplicons and their size in base pairs are indicated by brackets. (B) Agarose gel showing the obtained amplicons from L. amazonensis wild type (WT), ARG knockout (arg −) and the add-backs arg −/+ARG and arg −/+argΔSKL mutants genomic DNA templates with the indicated pairs of primers. (TIFF) [file pone.0034022.s003.tiff]

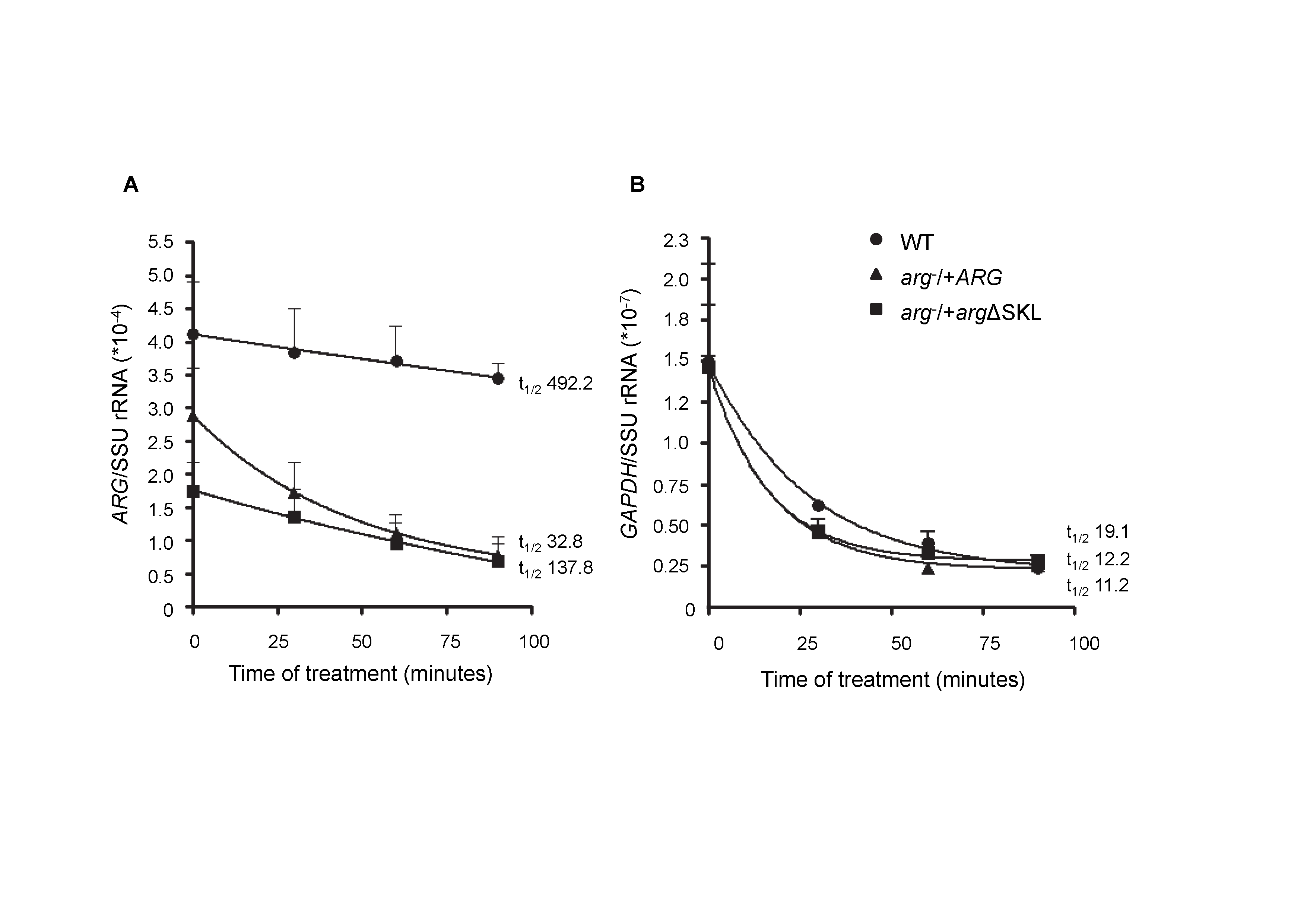

Supplement: Figure S3 — Half-lives of arg −/+ ARG and arg −/+ arg ΔSKL add-back parasites ARG mRNA are shorter than WT ARG mRNA. Relative copy-numbers of ARG (A) and GAPDH (B) mRNA, normalized by SSU rRNA expression, of L. amazonensis wild type (WT), ARG knockout (arg −), and the add-backs arg −/+ARG and arg −/+argΔSKL treated with actinomycin/sinefungin for mRNA decay determination. The obtained values are the means (+/− SD) of 3 independent experiments in duplicate. The black lines represent the exponential decay fitting with R2>0.98. t1/2 indicates the half-lives determined for each decay curve. (TIFF) [file pone.0034022.s004.tiff]

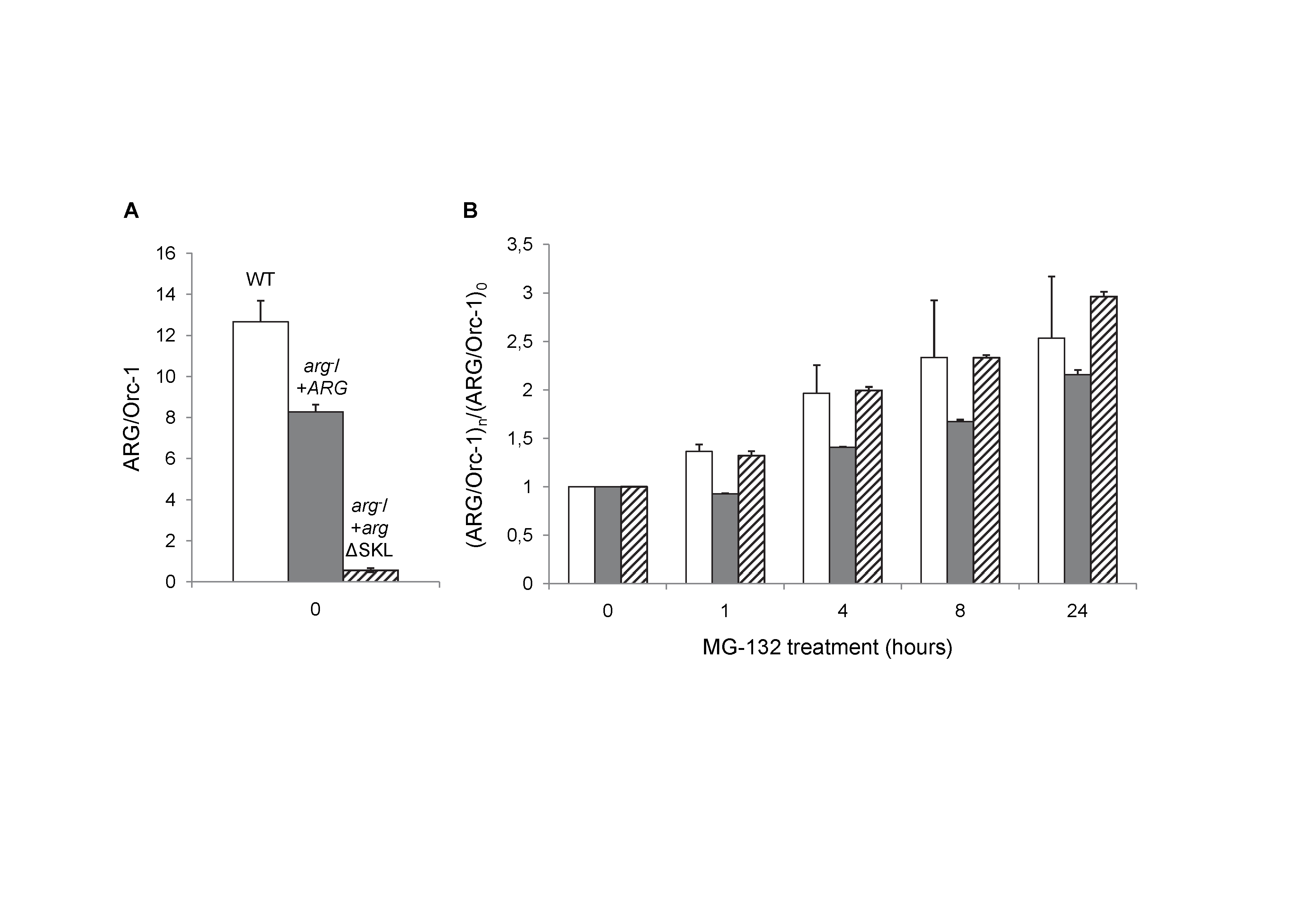

Supplement: Figure S4 — Proteasome inhibition with MG-132 causes ARG accumulation. ARG and Orc1 (loading control) levels in L. amazonensis wild type (WT, white bars), and the add-backs arg −/+ARG (gray bars) and arg −/+argΔSKL (dashed bars) were determined by western blot after 0 to 24 hours of treatment with MG-132 50 µM (Fig. 1C). (A) ARG quantification before MG-132 treatment. Values were normalized by Orc-1 quantification and are the means (+/− SD) of duplicates of a representative experiment. (B) The increase in ARG/Orc-1 levels after 1, 4, 8 and 24 hours of treatment with MG-132. ARG/Orc-1 values for each strain were normalized by ARG/Orc-1 level of the same strain before treatment. (TIFF) [file pone.0034022.s005.tiff]

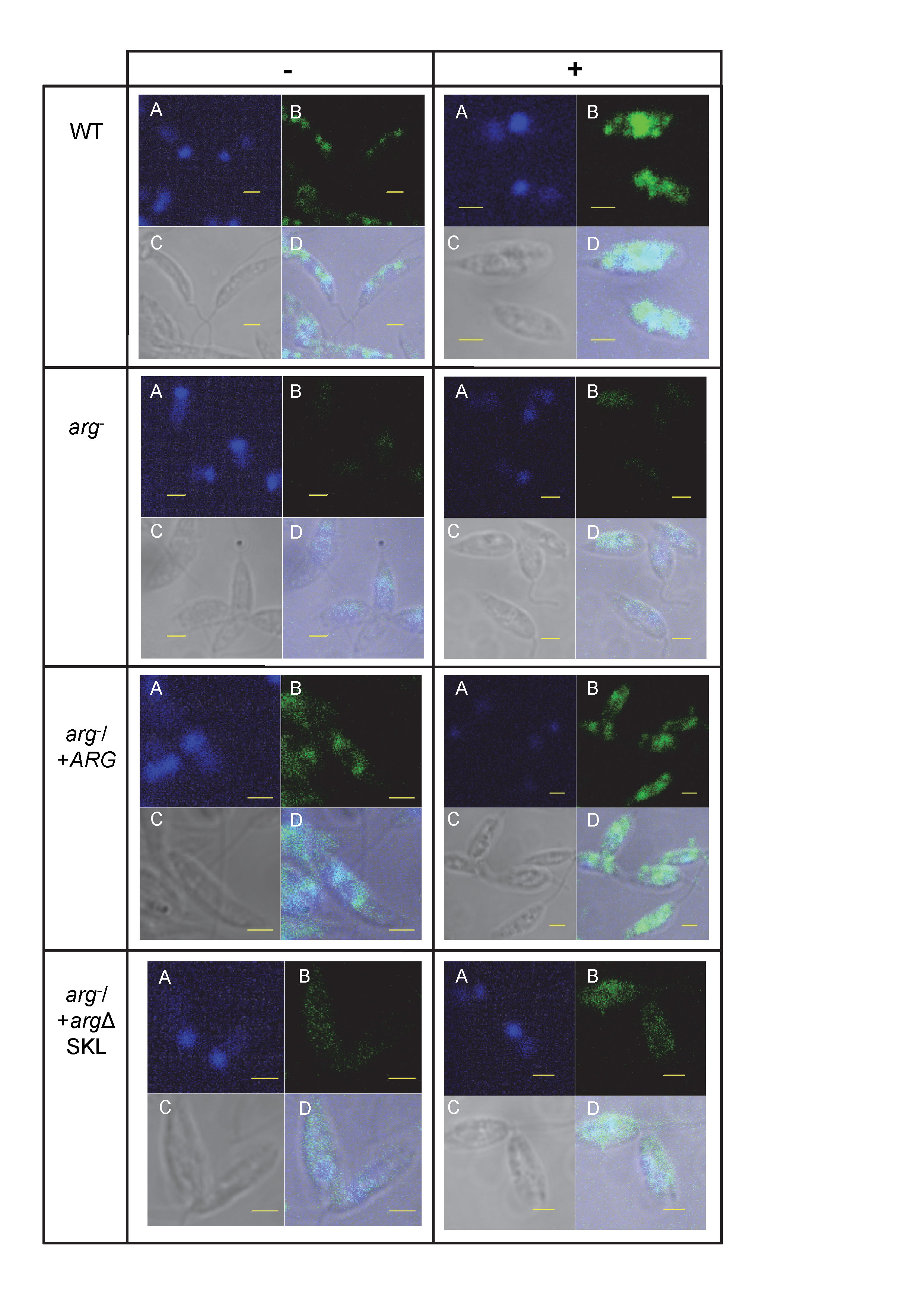

Supplement: Figure S5 — Inhibition of proteasome activity leads to ARG accumulation confirming cytosolic location in arg −/+ arg ΔSKL cells. L. amazonensis wild type (WT), ARG knockout (arg −), and the add-backs arg −/+ARG and arg −/+argΔSKL parasites were treated (+) or not (−) with 50 µM MG-132 during 24 hours and then submitted to immunofluorescence assays to determine ARG sub-cellular localization. (A) Blue labeling, nuclei staining with DAPI. (B) Green labeling, ARG immunolabeling. (C) Phase contrast. (D) Merge of A, B and C. Images are representative of at least 5 different fields. Scale bar: 2 µm. (TIFF) [file pone.0034022.s006.tiff]
