# Supplementary material for: Leishmania amazonensis Arginase Compartmentalization in the Glycosome Is Important for Parasite Infectivity
Source: PLoS One. 2012 Mar 30;7(3):e34022. doi: 10.1371/journal.pone.0034022 (PMC3316525; doi:10.1371/journal.pone.0034022)
Supplement: Table S1 — Nucleotide sequences of primers used. (DOCX) [file pone.0034022.s007.docx]

**Table S1. Nucleotide sequences of primers used**.

| Primer | | Sequence (5´ - 3´) |
| --- | --- | --- |
| 1 | ARG5's_725 | GAATTCTGCGCCTTTAGTGTTGCGTAG |
| 2 | ARG5'as_1269 | GGTACCGGGGCAAATTTTAATTCTCTGC |
| 3 | ARG3's_2609 | TCTAGATCGTTGGTGCGTTCCCTTTTC |
| 4 | ARG3'as_3150 | CTGCAGTTTTCGGGTTGTTTTCCAGG |
| 5 | DHFRsense2 | GGTACCGAATTCGAAAGCTCACCTCATTCC |
| 6 | ORFas2 | TCTAGAGCAGCTCAAATCACCACCACACAC |
| 7 | M13F | GCCAGGGTTTTCCCAGTCACGA |
| 8 | M13R | CCTGTGTGAAATTGTTATCCGCTC |
| 9 | 5L_BamHI_ARG | CCGGATCCTCATGGAGCACGTGCAGC |
| 10 | 3L_BamHI_ARG | GTGCGCACGGATCCGTCTACAGC |
| 11 | 3L_BamHI_ARGsSKL | ACGGATCCTAGCTCGTATGCGGAGTGTAA |
| 12 | ARG_5UTRs | TCGGGGTGATACGTTGGCCTTCAGTACGCATGCTTGTCTCCCTCC |
| 13 | ARG_3UTRas | GGAGCGCGAGAGTGAAGGAGAGCAAGAAACAACAGTGGTCGTGGTG |
| 14 | pHyg_s | CCGAGCACTAGCTAGTGATGAAAAAGCCTGAACTCA CCGCGACGT |
| 15 | pHyg_as | TTCGGTCGGCATCTACTCTATTCCTTTGCCCTCGGACGAGTGCTG |
| 16 | Hyg3S | GCTCCGGGCGTATATGCTCC |
| 17 | SMB 3545 | GGCCACCGTCGGCGTCTCGC |
| 18 | SMB 3546 | TGCGCGGTCCTTCGGGCACC |
| 19 | S1s | GATCTGGTTGATTCTGCCAG |
| 20 | S4as | GATCCAGCTGCAGGTTCACC |
| 21 | ARG22mod | GTGTCATACGACGTGGACACG |
| 22 | ARG_SC | CGACAAGACGACCGCACTCGGC |
| 23 | GAPDHrealF | CACACGTAGCCGCAGCTGG |
| 24 | GAPDHrealR | GCGTCCAGCACTGGAGCTAC |
